# Perinatal and laboratory determinants of fortified human milk use: a case-control study

**DOI:** 10.1590/1414-431X2025e14750

**Published:** 2026-02-16

**Authors:** C.B.G. Tavares, J.M.V. Zacarias, V.H. Souza, J.V. Visentainer, J.E.L. Visentainer

**Affiliations:** 1 Programa de Pós-Graduação em Biociências e Fisiopatologia, Universidade Estadual de Maringá, Maringá, PR, Brasil; 2 Departamento de Cadeiras Básicas, Faculdade de Medicina de Marília, Marília, SP, Brasil; 3 Departamento de Medicina, Universidade Cesumar (UNICESUMAR), Maringá, PR, Brasil; 4 Programa de Pós-Graduação em Ciências de Alimentos, Universidade Estadual de Maringá, Maringá, PR, Brasil

**Keywords:** Preterm infants, Low birth weight, Human milk, Dietary supplements

## Abstract

This case-control study compared perinatal and laboratory factors between preterm infants who received unfortified human milk (HM) and those who received HM fortified with FM85^®^. The sample included 38 low birth weight (LBW) preterm infants admitted to a neonatal intensive care unit between June 2022 and May 2023, excluding those with comorbidities or formula use. Perinatal variables including gestational age, birth weight, head circumference (HC), and mode of delivery were analyzed for associations with fortifier use using chi-squared and Student's *t*-tests. Laboratory values were compared across nutritional stages using the Kruskal-Wallis and Mann-Whitney tests. Very preterm infants (confidence interval [CI]: 4.02-21.10; P<0.001) and those with LBW (CI: 1.30-5.75; P=0.009) were more likely to receive the fortifier, particularly those with poor weight progression during trophic feeding (CI: 1.12-16.30; P=0.039). Altered HC (CI: 1.49-8.24; P=0.004) and cesarean delivery (CI: 2.24-11.30; P<0.001) were also associated with FM85^®^ use. Both groups showed laboratory abnormalities with neutrophils, initially elevated, decreasing (P<0.0001), while lymphocytes increased (P<0.0001). Red blood cell (P=0.00026) and hemoglobin levels (P=0.00007) worsened. Sodium and calcium levels rose significantly (P<0.0001 for both). HM fortification did not significantly alter most laboratory values, except for red blood cell count. LBW, altered HC, and cesarean delivery were associated with a greater likelihood of fortifier use. Immunological changes occurred even with fortification. Homologous fortifiers may represent a more physiological alternative.

## Introduction

Human milk (HM) contains more than 200 species-specific substances, such as immunoglobulins, organism-specific components like lactose, and both species- and organism-specific components like lipids. It also exhibits intrinsic variations depending on the type of milk, the nutritional status of the lactating mother, and the duration of lactation (1).

Breastfed infants receive both prebiotic and probiotic components (2), including bifidobacteria and lactobacilli from HM, which make up more than 90% of the saprophytic intestinal biome in newborns (3). Additionally, HM contains bifidogenic stimulators (prebiotics) such as species-specific proteins, lactoferrin, α-lactalbumin, lactose, nucleotides, and oligosaccharides (2).

HM is essential for children's health and is recommended for both full-term and premature babies. Most of its energy comes from glucose, which is converted into lactose. Long-chain polyunsaturated fatty acids like docosahexaenoic acid and arachidonic acid are part of its composition. It also contains essential proteins and amino acids that cannot synthesized by the infant. Therefore, HM is considered the best source of nutrition, providing the baby with adequate amounts of energy and nutrients (4).

Given the above, early use of expressed HM (EHM) from the infant's own mother is important for preventing complications in preterm infants (PTIs) that cannot breastfeed directly. If EHM is not available, pasteurized HM (PHM) is the best alternative (5). To meet this demand, HM banks operate in a standardized manner to ensure quality products for PTIs and those with low birth weight (LBW) (6).

The first 2 weeks of life are considered critical for PTI's growth and nutrition. These infants often lose weight between days 4 and 9 of life, after which growth rates usually replicate intrauterine development. Birth weight (BW) is typically regained in around 8 days for larger infants and close to 24 days for very LBW (VLBW) PTI's. To support recovery, it is a common practice to supplement HM with additives derived from bovine milk. In Brazil, the most commonly used commercial supplement is FM85^®^ (Nestlé, Brazil), which provides additional proteins, carbohydrates, vitamins, minerals, and trace elements to HM (7), but may expose the infant to components that can cause intolerances and allergies (8). Therefore, this study examined the differences between PTI's who received HM alone and those who received FM85^®^ fortified HM to indentify the advantages disadvantages of the two approaches not only from a nutritional standpoint, but also from a clinical perspective, using laboratory tests. In addition, the perinatal factors associated with supplementation were assessed.

## Material and Methods

An analytical case-control study was conducted in the neonatal intensive care unit (NICU) and HM Bank of the University Hospital (HUM) at the State University of Maringá (UEM).

### Inclusion and exclusion criteria

Preterm and LBW infants with gestational age (GA) between 28 and 35 weeks and BW between 1,000 and 2,499 g were included.

Infants with comorbidities and/or who received milk formulas, as well as those with extreme prematurity or very or extremely LBW (VLBW and ELBW), as these conditions can interfere with nutritional planning and affect study outcomes were excluded.

The included infants were divided into two groups: a control group (n=22) who received only HM, and a case group (n=16) who received FM85^®^-fortified HM, totaling 38 babies. HM fortification was performed according to the manufacturer's instructions (1 g per 25 mL of HM).

### Data sources

Laboratory test results were extracted from electronic medical records from the NICU. Perinatal variables were obtained from secondary records in the patients' charts.

### Perinatal variables

Variables collected were GA, BW, sex (male/female), head circumference (HC) (9), mode of delivery (cesarean or vaginal), maternal age, and parity.

GA was categorized as extreme prematurity (22 to <28 weeks), severe prematurity (28 to <32 weeks), and moderate to late prematurity (32 to <37 weeks) (10). BW was categorized as: LBW (<2,500 g), VLBW (<1,500 g), and ELBW (<1,000 g) (11). Maternal age was categorized as: adolescent (10-19 years), appropriate reproductive age (20-34 years), and advanced maternal age (AMA) (≥35 years) (12). Parity was categorized as primiparous (first birth) or multiparous (two or more births) (13).

### Laboratory variables

Tests included complete blood count and electrolyte levels. All infants underwent laboratory tests at three nutritional stages: fasting, trophic nutrition, and full nutrition (for controls) or FM85^®^-fortified nutrition (for cases). Trophic nutrition, or minimal enteral nutrition, was defined as 50-100 kcal/kg/per day. Full nutrition was defined as 110-150 kcal/kg/per day (9,11). This longitudinal nutritional comparison aimed to identify laboratory changes across nutritional stages. Samples were collected after a maximum of 24 h of fasting and 48 h after starting trophic, full, or FM85^®^ diets. Infants were monitored for an average of 12 days to reach all stages. During fasting, none of the infants received parenteral nutrition.

### Statistical analyses

First, perinatal variables were compared between the two using the chi-squared (χ^2^) test and Student's *t*-test. Next, laboratory test results were compared across nutritional stages using the Mann-Whitney and/or Kruskal-Wallis tests. A significance level of P<0.05 was adopted for all tests.

Perinatal variables such as GA, HC, and mode of delivery were examined for associations with the use of HM additives. Laboratory test results were compared between case and control groups from fasting through final nutrition. BW and GA were considered potential confounding factors, given the study population's characteristics (prematurity and LBW). Logistic regression was used to analyze the data and control for these factors when assessing associations between the groups.

The study was approved by the Ethics Committee (COPEP/UEM/5.257.520) of the State University of Maringá, and informed consent was obtained from the mothers or legal guardians.

## Results

The majority the 38 PTI's included in the study (57.90%) were female. Late and moderate PTIs accounted for 55.26% of the sample, while VPTIs comprised 44.74%. Regarding BW, 26.32% were classified as VLBW and 73.68% as LBW. Male infants showed greater alterations in HC (37.50%) than did female infants (27.27%). The cesarean section rate was 63.16%. Primiparous mothers represented 47.37% of the study population, and adolescent or AMA mothers accounted for 34.21%.

As shown in Table 1, male infants had a higher likelihood of FM85^®^ supplementation. A similar trend was observed for VLBW infants. Those who did not achieve expected weight gain during trophic and full nutrition phases also showed an increased likelihood of receiving FM85^®^. Additionally, infants with abnormal HC measurements were more likely to receive supplementation. VPTIs had a higher likelihood of receiving FM85^®^ than moderate and late PTIs, as did infants delivered by cesarean section. By contrast, infants born to AMA mothers were less likely to receive the additive. No statistically significant association was found between parity and FM85^®^ use.

**Table 1:** Characteristics of the whole sample and by group of preterm infants.

Variables	Total (%)	Control (%)	Fortified milk (%)	OR	95%CI	P	P*
Sex							
Female	22 (57.89)	16 (59.26)	6 (54.55)	1.0	(Reference)		
Male	16 (42.11)	11 (40.74)	5 (45.45)	1.2	0.29-4.98	0.79	0.999
Birth weight							
1500-2499 g	28 (73.68)	24 (88.89)	4 (36.36)	1.0	(Reference)		
1000-1499 g	10 (26.32)	3 (11.11)	7 (63.64)	14.0	2.51-77.99	0.003	0.002
Weight at trophic feeding							
1500-2499 g	33 (86.84)	24 (88.89)	9 (81.82)	1.0	(Reference)		
1000-1499 g	5 (13.16)	3 (11.11)	2 (18.18)	1.8	0.25-12.45	0.562	0.615
Head circumference (girls)							
Normal	13 (59.09)	9 (33.33)	4 (36.36)	1.0	(Reference)		
Altered	4 (18.18)	2 (7.41)	2 (18.18)	2.3	0.23-22.140.487	0.584	
Without measurement	5 (22.73)	-	-	-	-	-	-
Head circumference (boys)							
Normal	8 (50.00)	1 (3.70)	7 (63.64)	1.0	(Reference)		
Altered	5 (31.25)	2 (7.41)	3 (27.27)	0.2	0.01-3.37	0.273	0.510
Without measurement	3 (18.75)	-	-	-	-	-	-
Gestational age							
32-35 weeks	21 (55.26)	17 (62.96	4 (36.36)	1.0	(Reference)		
28-31 weeks	17 (44.74)	10 (37.04)	7 (63.64)	3.0	0.69-12.76	0.142	0.167
Mode of delivery							
Vaginal	14 (36.84)	11 (40.74)	3 (27.27)	1.0	(Reference)		
Cesarean	24 (63.16)	16 (59.26)	8 (72.73)	1.8	0.40-8.49	0.438	0.488
Parity							
Primiparous or Secundiparous	24 (63.16)	19 (70.37)	5 (45.45)	1.0	(Reference)		
Multiparous	14 (36.84)	8 (29.63)	6 (54.55)	1.9	0.45-7.97	0.389	0.552
Maternal age							
Appropriate	26 (68.42)	19 (70.37)	7 (63.64)	1.0	(Reference)		
Adolescent	6 (15.79)	3 (11.11)	3 (27.27)	2.7	0.44-16.75	0.282	0.346
AMA	6 (15.79)	5 (18.52)	1 (9.09)	0.5	0.05-5.50	0.605	0.999
Total	38 (100)	27 (100)	11 (100)				

HC: head circumference; AMA: Advanced Maternal Age. *Results after logistic regression.

After logistic regression, infants with poor weight progression during trophic nutrition remained more likely to receive supplementation (odds ratio [OR]=3.97; confidence interval [CI]: 1.12-16.3; P=0.039). However, this association did not persist when weight gain was inadequate during full nutrition. Altered HC remained a significant risk factor (OR=3.44; CI: 1.49-8.24; P=0.004), and cesarean delivery further increased the likelihood of additive use (OR=4.85; CI: 2.24-11.3; P<0.001). AMA continued to be a protective factor against FM85^®^ supplementation (OR=0.18; CI: 0.07-0.43; P<0.001). Interestingly, male sex, initially a risk factor, emerged as a protective factor in the regression analysis (OR=0.42; CI: 0.19-0.90; P=0.030).

The Kruskal-Wallis test, used to compare laboratory values between case and control groups from fasting through full nutrition (or FM85^®^ nutrition), showed that elevated neutrophil counts during fasting decreased to acceptable levels (P<0.00001), accompanied by an increase in lymphocyte proportions (P<0.00001). Sodium and calcium levels also rose significantly (P<0.00001). Chloride levels initially increased but later returned to baseline fasting values (P<0.00001). Red blood cell counts (P=0.00026) and hemoglobin levels (P=0.00007) decreased (Table 2). The FM85^®^ diet influenced red blood cell production, supported by statistically and biologically consistent evidence (P=0.0083). However, FM85^®^ use did not significantly alter other clinical laboratory parameters (Table 3), thus larger sample sizes are needed to draw more definitive conclusions.

**Table 2:** Comparison of laboratory analyses between premature infants at different nutritional stages fed human milk and fortified human milk.

Parameters	Fasting stage		Trophic feeding	Full feeding	P-value
Red blood cells (million/µL)	4.4 (3.5-5.3)		4.1 (3.1-5.1)	3.8 (3.3-4.3)	**0.00026**
Hemoglobin (g/dL)	16.1 (12.9-19.3)		14.7 (10.5-18.9)	13.1 (9.9-16.4)	**0.00007**
Glucose (mg/dL)	62.8 (16.1-109.6)		74.9 (56.2-93.7)	70.4 (45.4-95.4)	0.0784
Sodium (mmol/L)	132.3 (128.6-136.1)		136.3 (132.3-140.3)	134.5 (132.0-137.0)	**<0.00001**
Potassium (mEq/L)	4.3 (3.6-5.0)		4.2 (3.6-4.8)	4.1 (3.6-4.7)	0.43048
Calcium (mg/dL)	4.7 (4.2-5.2)		5 (4.6-5.4)	5.2 (5.0-5.4)	**<0.00001**
Chloride (mEq/L)	107.2 (104.2-110.2)		111.6 (108.6-114.6)	106.9 (102.9-110.9)	**<0.00001**
Leukocytes (/mm^3^)	13864.4 (7729.4-19999.4)		10187.1 (4887.4-15486.9)	11825.5 (8249.5-15401.5)	0.15549
Neutrophils (%)	65.6 (56.4-74.9)		43.1 (25.6-60.6)	39.7 (22.2-57.2)	**<0.00001**
Band neutrophils (rods) (%)	3.2 (-1.8-8.2)		1 (-1.0-3.0)	0.7 (0.2-1.2)	**0.0014**
Eosinophils (%)	0.6 (-0.4-1.6)		4.1 (2.1-6.1)	3.7 (0.2-7.2)	**<0.00001**
Basophils (%)	0.5 (-0.5-1.5)		0.8 (-0.2-1.8)	0.5 (0.0-1.0)	0.5648
Lymphocytes (%)	23.5 (11.3-35.8)		38.8 (20.6-57.1)	46.1 (26.6-65.6)	**<0.00001**
Monocytes (%)	9.9 (3.9-15.9)		13.2 (6.5-20.0)	9.4 (5.9-12.9)	**0.00175**

Data are reported as medians and interquartile range. Kruskal-Wallis test. Bold type indicates statistically significant.

**Table 3:** Laboratory test results of infants receiving unfortified (control group) and fortified (case group) human milk (HM).

Parameters	Unfortified HM	Fortified HM	Reference range**	P-value
Red blood cells (million/µL)	3.95±0.72	3.23±0.70*	3.9-6.3	0.0083
Hemoglobin (g/dL)	13.44±2.64	11.51±3.20	10.0-20.5	0.5744
Glucose (mg/dL)	72.46±16.60	71.36±13.53	40-80	0.6744
Sodium (mmol/L)	134.56±5.16*	131.64±2.01*	136-145	0.5744
Potassium (mmol/L)	4.08±0.42	4.35±0.40	3.7-5.9	0.1388
Calcium (mg/dL)	5.14±0.51	5.17±0.21	4.6-5.3	0.4715
Chloride (mmol/L)	106.7±3.68	105.45±2.31	96-110	0.1675
Leukocytes (/mm^3^)	11,739.04±5076.54	11,935.45±3327.11	5000-20,000	0.7263
Neutrophils (%)	38.56±11.74	40.73±14.12	20-50%	0.7718
Rods (%)	0.63±1.13	0.36±0.64	0-11%	0.7948
Eosinophils (%)	3.59±3.15	5.45±5.25*	1-4%	0.3953
Basophils (%)	0.52±0.74	0.36±0.64	0-1%	0.6312
Lymphocytes (%)	46.44±13.54	43.55±14.33	25-85%	0.7278
Monocytes (%)	10.15±4.18	9.82±2.76	1-14%	0.7489

Data are reported as means and SD. Student's *t*-test. *Values outside the reference range. **Reference range for newborns aged 0-4 months.

## Discussion

In this study, there was a higher number of female infants than male. According to some studies (14,15), mothers in poorer conditions tend to give birth to girls, while those in better conditions tend to have more boys. Prenatal stress can increase male fetus vulnerability and contribute to poor fetal outcomes. The samples in this study were collected between 2022 and 2023, during the COVID-19 pandemic, suggesting that maternal stress may have influenced the higher proportion of female births. However, while some studies (15-17) support this observation, others do not. Therefore, more detailed research is needed to determine whether the pandemic had a real effect on sex distribution.

Initially, male sex was identified as a risk factor for supplementation, but after logistic regression, it emerged as a protective factor. A study on VLBW preterms also found no sex-based differences in the risk of supplementation (7), likely because nutritional guidelines for PTIs are designed to support growth and development regardless of sex (18).

In Brazil, the estimated prematurity rate is 11.50%. Late PTIs make up the majority (74%), followed by those with ≤31 weeks (16%) and those with 32-33 weeks (10%) (16). In this study, 55.26% were moderate and late PTIs (GA between 32-35 weeks), and 44.74% were VPTIs (GA between 28-31 weeks), with the latter showing a higher risk of requiring additives, consistent with established protocols (18,19).

These proportions differ from national averages, likely because HUM is a referral center for high-risk pregnancies, with a high prevalence of mothers at age extremes and prenatal care provided during the pandemic. As part of the Unified Health System, the hospital serves a population with unfavorable socioeconomic conditions and therefore more vulnerable to adverse perinatal outcomes (19).

PTIs weighing between 1,000-1,499 g had a higher likelihood of requiring supplementation, and this risk increased when LBW persisted during trophic feeding. A child's health is directly tied to intrauterine weight gain and its maintenance after birth; therefore, identifying the contribution of prematurity and intrauterine growth restriction to LBW is essential for preventing the condition, meeting nutritional needs, and addressing its health consequences, especially in this population at higher risk of morbidity and mortality (8,20).

Studies suggest that a diet with additives may interfere in weight and linear growth, even though the fat content in HM from mothers of PTI's is higher during early lactation than in later stages (7,20,21). The recommended intake for ELBW PTI's is 4.0-4.5 g/kg/day of protein and 110-135 kcal/kg/day; however, the natural composition of HM contains lower concentrations of both, highlighting the need to supplement these nutrients (7,18,22).

One solution is supplementation with heterologous additives, which enriches HM with additional nutrients (8,18,19). However, this approach may alter immune protection, affect intestinal maturation, and lead to dietary intolerances (23). Moreover, the combination of HM and additives increases osmolarity, potentially resulting in protein deficiency or overload (7). Accelerated weight gain from supplementation has also been linked to insulin resistance and increased adiposity (24).

One option during this complex stage where standardized supplementation may pose risks is the use of homologous additives for PHM or EHM (8,23,25). While bovine milk protein is more readily available, its use may contribute to the development of future diseases (24,26). It is important to emphasize that the exclusive use of HM reduces infection rates and supports improved neurological development (7,8,23). As such, homologous additives that provide higher-quality amino acids and functional fatty acids are currently being tested (25).

PTIs with altered HC (9) and those who failed to progress during trophic feeding received more FM85^®^ supplementation. Because HC reflects brain development, values outside the ideal range are considered unfavorable for neurodevelopment (27). In PTIs, HC should increase by 0.5-0.8 cm per week. Nutritional support strategies for PTI growth and development are guided by established growth curves (9).

Babies fed HM show better performance on intelligence tests and may attain higher educational levels and income in adulthood (28). This further supports the use of EHM or PHM and, when necessary, homologous additives especially since there is limited evidence that fortified HM significantly impacts long-term outcomes beyond modest gains in in-hospital growth (29).

Cesarean delivery was also identified as a risk factor for the use of additives in HM. According to the World Health Organization, cesarean rates above 15% suggest overuse of the procedure. In 2019, Brazil's cesarean rate reached 56.3%, with even higher rates observed in private hospitals (30).

A study conducted in Porto Alegre, Brazil, which examined the relationship between cesarean delivery and prematurity, highlighted the impact of prematurity through its association with lower Apgar scores and LBW. Prematurity and LBW are major contributors to infant mortality, largely due to poor nutrition and metabolic immaturity (31). Targeted actions to reduce cesarean rates are essential to help reduce prematurity rates, LBW, and maternal and neonatal mortality (30).

Parity did not show significant associations with the use of HM additives. However, other studies (12,20) have reported a relationship between parity, prematurity, and adverse perinatal outcomes in various populations, highlighting the need for improved health policy development tailored to specific population profiles (30).

AMA emerged as a protective factor against the use of FM85^®^_,_ although babies of both older adult and adolescent mothers had a higher likelihood of fortifier use. The rates of mothers at age extremes were higher than national averages, likely because HUM is a referral center for high-risk pregnancies, as previously discussed.

In Brazil, adolescent and older adult mothers face a greater risk of prematurity due to inadequate or delayed interventions during pregnancy and postpartum. As a result, public policies should prioritize timely and appropriate care during the prenatal and postnatal periods (12), while also supporting and protecting breastfeeding, preserving lactation, and enabling parental presence in the NICU. These measures can lead to better outcomes in health, education, cognitive development, and future earning potential (28,31).

Glucose levels did not show significant associations with the use of fortifiers. However, VPTIs and extremely premature infants may experience glucose intolerance due to reduced insulin sensitivity and elevated counter-regulatory hormones triggered by stress. Glucose is particularly important in this context because PTIs have limited glycogen stores and require 25-50% of their caloric intake from carbohydrates. In HM, the main carbohydrate is lactose, a disaccharide composed of glucose and galactose (18).

According to the package insert, 100% of the carbohydrate content in FM85^®^ is maltodextrin. This oligosaccharide undergoes hydrolysis in the gastrointestinal tract, and its absorption leads to a rapid glycemic response. The increase in osmolarity in HM is due to both the addition of supplements (which increase solutes) and the activity of HM amylase, which, when activated, breaks down dextrins into monosaccharides and oligosaccharides, further raising osmolarity (8,23,29,32).

Given this, glucose intake is necessary to provide energy and compensate for the metabolic deficiencies of PTIs. However, a diet based on HM has lactose as its main carbohydrate, supplying 45-50% of total energy, in addition to free glucose (0.02 g/L), glycolipids, nucleotides, glycoproteins, and oligosaccharides. Thus, HM provides essential nutrients, including glucose, without posing a risk of necrotizing enterocolitis (1,23).

The infants in this study showed alterations in chloride levels, as well as in sodium and calcium levels throughout nutrition. Hyperchloremia and hypernatremia are common in critically ill children. A study on children in similar conditions found 8.6% hyperchloremia, 2.6% hypochloremia, 16% hypernatremia, and 10% hyponatremia, with 3.3% presenting both hypernatremia and hyperchloremia upon admission (33). The diet can influence these values both positively, when balanced, and negatively, with the unnecessary use of additives. The macrominerals present in HM (potassium, chloride, calcium, sodium, phosphorus, magnesium) help maintain plasma osmolarity close to physiological levels (1).

Inadequate nutritional support can lead to complications, but HM remains the best nutritional choice. However, its nutrients are often considered insufficient to meet the needs of PTI (18). Therefore, homologous fortifiers would be an ideal alternative to address this deficiency (8,23,25).

Anemia of prematurity results from the immaturity of the hematopoietic system, iatrogenic blood loss, erythropoietin deficiency, substantial growth rates, and underlying diseases. Hemoglobin levels are lower in PTIs due to decreased erythropoietin production. In these infants, fetal hemoglobin has a higher affinity for oxygen (97%), making oxygen delivery to tissues more difficult (34). The observed reduction in red blood cell and hemoglobin counts in both groups suggests a potential interference of the FM85^®^ diet in red blood cell production, supported by consistent statistical and biological evidence.

Preventing anemia in PTI is critical, and early iron supplementation is a key preventive measure. The iron concentration in the HM of mothers of PTIs is sufficient to prevent anemia, with a positive correlation between lactation duration and iron levels (24,34). Therefore, the use of EHM should be encouraged to prevent anemia without the need for additives.

The elevated neutrophil count observed during fasting decreased to acceptable levels, replaced by a higher proportion of lymphocytes during full nutrition. In addition to being a rich source of nutrients, HM contains immunological factors such as enzymes, immunoglobulins, cytokines, complement system components, and leukocytes, providing PTIs with passive immunity and immunomodulation. These agents are resistant to digestive enzymes, protect mucosal surfaces, and eliminate bacteria without causing inflammation (3).

The comparison of laboratory tests between groups showed that supplementation with FM85^®^ did not affect the results. The immunity provided by HM remains unaffected by pasteurization (5,6,35) or supplementation, supporting the superiority of this product, particularly for PTIs who are more susceptible to infections. HM offers ideal nutrition for PTIs while fostering significant immunological interaction between mother and child (3).

LBW, altered HC, and cesarean section were identified as predisposing factors for the use of FM85^®^. These findings underscore the importance of high-quality prenatal and delivery care to mitigate adverse perinatal outcomes.

The data also suggest a potential interference of the FM85^®^ diet in red blood cell production. Overall, supplementation with FM85^®^ did not positively impact the laboratory results. Immunological changes, such as decreased neutrophils and increased lymphocytes, were observed even with fortified HM, but other factors may also influence leukocyte counts in PTIs. Additionally, other laboratory findings showed trends that could be relevant, but larger studies are needed to draw more definitive conclusions about their specificity. These two factors (potential influences from other variables on leukocytes and the need for larger sample sizes) are limitations of this study. Given these findings, homologous fortifiers are being investigated as a possible future alternative to bovine-derived fortifiers, offering a more physiological approach.

## Data Availability

All data generated or analyzed during this study are included in this published article.
